# Sociodemographic and behavioural factors associated with body mass index among men and women in Nairobi slums: AWI-Gen Project

**DOI:** 10.1080/16549716.2018.1470738

**Published:** 2018-07-03

**Authors:** Gershim Asiki, Shukri F. Mohamed, David Wambui, Caroline Wainana, Stella Muthuri, Michelle Ramsay

**Affiliations:** a Health and Systems for Health Unit, African Population and Health Research Center, Nairobi, Kenya; b Department of Women’s and Children’s Health, Karolinska Intitutet, Stockholm, Sweden; c Sydney Brenner Institute for Molecular Bioscience, Faculty of Health Sciences, University of the Witwatersrand, Johannesburg, South Africa; d Division of Human Genetics, National Health Laboratory Service and School of Pathology, Faculty of Health Sciences, University of the Witwatersrand, Johannesburg, South Africa

**Keywords:** Body Mass Index (BMI), urban slums, Nairobi, Kenya, associated factors

## Abstract

**Background**: Body mass index (BMI) is rising globally with a faster increase in urban areas in low- and middle-income countries. It is critical to identify modifiable risk factors for BMI to prevent the occurrence of associated health consequences.

**Objective**: To investigate socio-demographic, behavioural and biological factors associated with BMI in Nairobi slums.

**Methods**: In 2014-2015, a cross sectional study of men and women aged 40–60 years in Nairobi slums (Korogocho and Viwandani) was conducted. Data were collected on socio-demographic, behavioural and biological characteristics. Mean BMI, overweight and obesity were computed. Hierarchical multiple linear regression analysis was conducted separately for men and women to explore factors associated with BMI.

**Results**: In total, 1942 study participants (54.4%, women) with a mean age (SD) of 48.3 (5.3) years and 48.8(5.6) years for women and men respectively were recruited. Mean BMI was higher among women than men (27.6 versus 22.8; p < 0.001). More women were overweight (30.9% versus 19.6%; p < 0.001) and obese (32.1% versus 5.1%; p < 0.001) than men. Among men, BMI was independently associated with wealth index, bread consumption and self-reported diabetes and was negatively associated with current tobacco smoking, HIV and TB infections. Among women, BMI was independently associated with wealth, current non-problematic drinking, and sedentary time, but was lower among other ethnicities compared to Kikuyu, among current smokers, women with longer sleep, and those with HIV infection and tuberculosis. Wealth index contributed the most variance in BMI among women and men (10.4%, 7.5%, respectively), but behavioural factors (7.4%) among men and biological factors (6.5%) among women accounted for most of the additional BMI variance.

**Conclusions**: Adults aged 40–60 years in the urban slums of Nairobi have a high BMI associated with wealth. Bread consumption by men and sedentary life among women are the main risky behaviours that need urgent targeted interventions.

## Background

In the 2017 World Health Organization report, it was estimated that more than 2.5 billion adults globally had a high Body Mass Index (BMI >25 kg/m^2^); 1.9 billion were classified as overweight and 650 million as obese [1]. Obesity has tripled over the last four decades with a faster increase in low- and middle-income countries (LMICs) mainly attributed to a rapid urbanization [1]. It is estimated that by 2025, three quarters of the obese population will live in non-industrialized countries [2]. The rise in overweight and obesity is particularly worrying because of the associated health consequences such as cardiovascular diseases, diabetes, cancers and osteoarthritis [3]. It is projected that by 2030, more than 50% of Africa’s population will be living in urban areas [4], with majority living in slums where access to social services including health is limited [5]. An analysis of Demographic and Health Survey (DHS) data from seven countries in Sub-Saharan Africa (SSA) including Kenya showed that the prevalence of urban overweight and obesity increased by nearly 35% between 1992 and 2005 [6].

The first nationally representative survey to collect comprehensive information on risk factors for NCDs in Kenya in 2015, revealed that 27% of Kenyans were either overweight or obese with 32% prevalence among urban and 23% among rural residents respectively [7]. In Nairobi, the capital city of Kenya, where 60% of the population is estimated to be living in slums or slum-like conditions [8], 43.4% of women and 17.3% of men were overweight or obese [9]. Given the high poverty levels in the slums, most of the population may not have financial resources and knowledge to adapt healthier life styles and access treatment for NCDs. For example, in this study among adults aged 40 to 60 years, hypertension prevalence was 25.6% in the slums of Nairobi: 58.9% of men were not aware of their hypertension status, 28.2% of women were not aware and only 50% of women and 30% of men had controlled blood pressure [10]. Kimani et al, previously reported high levels of catastrophic health expenditure among slum residents with up to 90% of the population not accessing any form of health insurance [11]. Overweight and obesity are largely preventable. Identifying the modifiable risk factors for overweight and obesity may provide important insights into the design of focused intervention strategies that would help avert unaffordable costs of treatment for NCDs in the slum population. Few studies have examined the risk factors for high BMI in urban slums. Epidemiological studies in Nairobi slums have focused mainly on obesity prevalence, with little emphasis on the contribution of socioeconomic, behavioural and biological as risk factors for obesity [9,12], and the risk factors for high BMI among men and women have not been separately examined in the same population yet, wide gender variations may exist in the risk factors. A study among women in Nairobi slums showed that most of the variance in BMI was explained by age, total physical activity, and percentage of fat consumed, parity and socio-economic group, together accounting for 18% of the variance in BMI [13]. No corresponding study examined the contribution of these risk factors among men.

**Figure 1.**
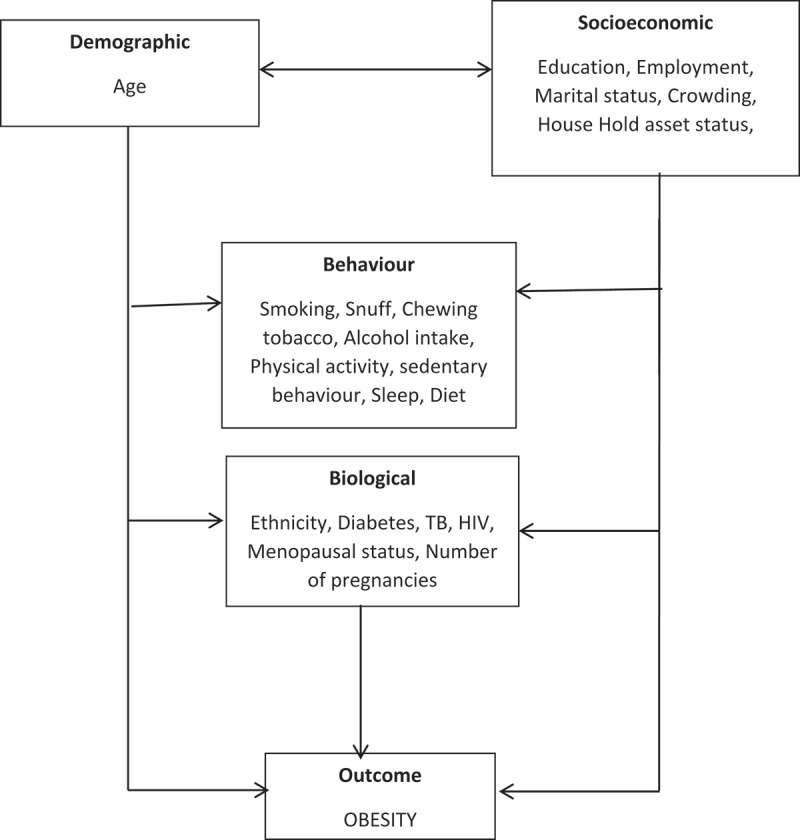
A theoretical conceptual framework of risk factors for obesity.

In this paper, our main aim was to investigate the influence of socio-demographic, behavioural and biological level correlates on BMI using multi-level techniques to elicit complex hierarchical relationships separately for men and women. As shown in Figure 1, we hypothesized that sociodemographic factors (age, education, employment, marital status, crowding, wealth status) may affect directly or indirectly all other risk factors such as behavioural factors (smoking, snuff, chewing tobacco, alcohol intake, exercise, sedentary behaviour, sleep, diet) and biological factors (ethnicity, diabetes, tuberculosis, HIV, menopausal status, and number of pregnancies). These factors may in turn affect obesity independently. The ultimate aim was to identify the contribution of these risk factors to high BMI to tailor gender-specific interventions.

## Methods

### Study design and setting

This was a cross-sectional population-based study conducted in 2014-2015 in two urban slums of Nairobi as part of a multi-country study through a partnership between University of the Witwatersrand (Wits), Johannesburg, and the International Network for the Demographic Evaluation of Populations and Their Health (INDEPTH), referred to as the Africa Wits-INDEPTH partnership for Genomic Studies (AWI-Gen) [14]. The primary aim of the AWI-Gen study is to explore genetic and environmental factors that contribute to cardio-metabolic disease in African populations. The Nairobi Urban Health & Demographic Surveillance System (NUHDSS) in Kenya [15], where this study was conducted is one of six AWI-Gen sites in four Sub-Saharan African countries [14]. The NUHDSS, the first urban-based HDSS in SSA, was established in 2002 currently following a population of about 75,000 individuals in approximately 24,000 households in Korogocho and Viwandani slums in Nairobi [15].

### Study population and sampling

Adults registered as residents in the NUHDSS aged 40–60 years were included in the study. The choice of this age group was informed by the high prevalence of cardiometabolic diseases risk factors from the age of 40 years and a small number of adults above 60 years in the slums. Pregnant women and individuals with physical impairments preventing measurement of blood pressure and other anthropometric indices were excluded from the study. Using the NUHDSS census database of 2014-2015, adults aged 40–60 years were randomly selected from the database and invited to a central location for screening. Those who never met the inclusion criteria or declined to participate were replaced through a random selection of additional participants until a sample size of 2000 was accrued.

### Data collection and management

Trained field interviewers administered a paper questionnaire to participants with questions on socio-demographic characteristics (age, education, employment, marital status, crowding, wealth status), behavioural characteristics (smoking, snuff and chewing tobacco, alcohol intake, physical activity, sedentary behaviour, sleep, diet) and biological factors (ethnicity, diabetes, tuberculosis, HIV infection, menopausal status, and number of pregnancies) as explanatory variables. Anthropometry (weight and height) were measured to compute BMI as the outcome variable.

Data were entered into a Research Electronic Data Capture (REDCap) computerized database [16]. For quality control purposes, 10% of all entries were checked for consistency between the paper form and the electronic versions. Anonymized data were then transferred though a secure File Transfer Protocol connection to the central REDCap server at the University of the Witwatersrand, where a second process searched for outliers and missing data. After completion of the quality control process, the data were securely stored.

### Measurements and definitions of explanatory variables

*Marital status*: The marital status of participants was categorized as follows: never married or co-habited; married or living with partner; divorced/widowed.

*Household density*: This was calculated as the number of people in a given household divided by the number of bedrooms in the house.

*Wealth index*: Information was collected on ownership or possession of various household items such a television, radio, refrigerator, cooker (with oven), sofa set, microwave, home computer, mobile phone, landline, land/plot, livestock, and a vehicle. Wealth index was calculated using the principal component analysis method commonly used in Demographic and Health Surveys (DHS) Program [17].

*Tobacco, alcohol use and diet*: A questionnaire was administered to assess use of smoking tobacco products, and smokeless tobacco such as chewing tobacco and/or snuff tobacco, and each were categorized as follows: never used; current user; former user. The number of cigarettes or times a tobacco product were consumed daily was recorded. Alcohol use and potential abuse was determined according to the CAGE questionnaire [18]. Show cards were used to define a standard drink and problem drinking was assessed by four CAGE questions among those who reported drinking. If participants felt they should cut down on their drinking or were annoyed by people criticizing their drinking or ever felt bad or guilty about their drinking or ever had an alcoholic drink first thing in the morning to steady their nerves or get rid of a hangover, they were classified as problem drinkers. Alcohol use was therefore categorized into: never consumed; current non-problematic consumer; current problematic consumer; former consumer. Standard show cards were also used to estimate the number of fruits/vegetables servings and slices of bread consumed per day.

*Moderate to vigorous intensity physical activity (MVPA) and sedentary life*: The Global Physical Activity Questionnaire (GPAQ) was used to obtain information on self-reported physical activity [19]. This tool was developed by the World Health Organization and has been validated for physical activity surveillance in developing countries. Total moderate-vigorous physical activity (MVPA) in minutes per week were calculated from the accumulation of occupation, travel-related and leisure time physical activity.Total sitting time (minutes/week) at work, or working on a computer, while watching TV or travelling, socializing or relaxing both during the week and on weekends was used as a proxy for sedentary behaviour. To estimate total hours spent sleeping, participants were asked when they would go to bed and wake up during the week and on weekends.

*Menopause*: Women were asked whether they had irregular periods and when they had their last period. Menopause was staged according to the guidelines of the North American Menopause Society [20] limited to observation of the participants’ menopausal cycle, thus women were categorized according to their menopausal status as pre-menopausal for those having current regular periods; peri-menopausal as having irregular periods or spotting within the past year; post-menopausal as having no period within the past year.

*Diabetes, tuberculosis and HIV*: Diabetes and tuberculosis prevalence were estimated based on self-reported questions. Participants were asked if they were told by a professional health worker that they had diabetes or tuberculosis. Whereas rapid HIV testing was conducted on all samples to ascertain HIV infection.

### Outcome measurement

Weight was measured using digital Physician Large Dial 200kg capacity scales (Kendon Medical) with participants wearing no shoes, heavy clothing, and jewellery and recorded to the nearest kilogram. Height was measured using a Harpenden digital stadiometer (Holtain, Crymych, Wales) in a straight posture with barefoot, or wearing thin socks and recorded in millimetres. Body mass index (BMI), the main outcome measure for this study was computed as a continuous variable.

### Validity and reliability

All instruments were calibrated, and protocols tested before the start of the study. Field workers, questionnaires and physical measurements for volunteer participants were checked and their results compared with those of an expert measurer; and ensured that all results were within a 5% margin of error. Every four months, field-workers were re-trained, and their technical skills and ability re-evaluated to ensure efficiency and consistency in data collection and management. Evaluation of each field worker was performed on all aspects including; consent and questionnaire administration, anthropometric measurements and intra- and inter-variability on 15 successive volunteer participants. Four precision estimates were calculated: the technical error of measurement (TEM), the relative technical error of measurement (rTEM), the coefficient of reliability (R) and the coefficient of variation (CV). An evaluation was reported as satisfactory if the intra- and inter- coefficient of variations were below 2–5%.

### Statistical analysis

Descriptive analyses were performed using proportions, means, and confidence intervals. Normally distributed continuous variables were described as mean and standard deviation, and non-normally distributed variables were described as median and interquartile range. Since BMI was not normally distributed, log transformation was performed. To explore associations with BMI, first a univariate linear regression was conducted, from which factors with p value less than 0.20 were included in a model. The selection of the factors was not based purely on statistical tests but a theoretical conceptual frame work (Figure 1) as proposed by Victoria et al. [21], since more traditional level p values such as 0.05 used to select variables can fail in identifying variables known to be important [22]. Variables were grouped into socio-demographic, behavioural and biological categories. Hierarchical multiple linear regression analysis was conducted separately for men and women. An initial model only including sociodemographic factors was built. All sociodemographic factors for which the association reached p ≤ 0.20 were included in a multivariate model. Factors that remained independently and significantly associated with the outcome (p ≤ 0.20) were retained. Next, the association between each determinant in the sociodemographic factors group, behavioural factors group, and biological factors were assessed by adding the single determinant into the multivariate model, including the subset of independently significant sociodemographic factors. A multivariate model was built that included the subset of socio-demographic factors in the first multivariate model plus behavioural or biological factors for which the p-value was 0.20 after adjusting for the socio-demographic factors. The final model was selected on the basis of theoretical framework and best fit (Bayesian Information Criterion-BIC). Statistical analyses were performed using STATA, version 13 (StataCorp, College Station, TX, USA).

## Results

### Characteristics of the study population

Of the 2003 participants recruited in the study, 1942 study participants (54.4%, women) who had no missing data were included in the analysis. The mean age and standard deviation was 48.5 (5.6) years for men and 48.3 (5.3) years for women with about one-third of the sample from Kikuyu ethnicity, the rest being constituted by Kamba (19.8%), Luhya (18.5%), Luo (16.3%) and other ethnic groups (9.5%). Nearly half of the women were from the Kikuyu ethnic group. Almost all men (91.2%) in the study were married or cohabiting while 46.1% of women were in marriage/cohabitation and 47.3% of women were divorced or separated or widowed. More than half of the women were either peri-menopausal or menopausal. Generally, more men were educated than women: only 3.8% of men had no formal education while 10.7% of women had no formal education. Up to 94.1% of the study population had some form of employment with slightly more men employed compared to women (97.4% versus 91.5%). Regarding household socioeconomic status, approximately one third of men were in the fifth wealth quintile while most women were in the second to fourth wealth quintile. Women on average reported five lifetime pregnancies. An average household density of about three people per room was reported and women lived in higher-density households than men (3.2 versus 2.3).

We also assessed NCD behavioural risk factors and found that over half of the men had used tobacco as current smoker (22.8%), former smoker (27.2%), and snuff user (3.5%) and chewing (0.5%) while less than 10% of women reported tobacco use; 2.2% were current smokers, 4.9% were former smokers, 1.1% snuff users and 0.4% tobacco chewers. Alcohol consumption was also higher among men than women: Current non-problematic (19.6% versus 3.0%), current problematic (14.2% versus 2.8%) and former consumer (37.1% versus 22.4%) among men and women, respectively. Men reported more time in moderate-to-vigorous physical activity than women while women reported slightly more night hours of sleep than men. Sedentary time did not vary by sex. Regarding dietary habits, the overall fruit and vegetable consumption was below the recommended five servings per day, although women consumed more vegetable servings than men. Men consumed more slices of bread, sugary drinks and ate food out or from a vendor. Diabetes (4.1% versus 2.0%), HIV infection (16.2% versus 7.0%) were reported to be higher among women than men while there was no statistically significant difference in tuberculosis infection (10.7% versus 11.2%) between women and men (Table 1).

**Table 1.** Distribution of socio-demographic, behavioural and biological characteristics by sex.

Characteristics	Men	Women
**Sociodemographic**		
Sex	886 (45.6%)	1056 (54.4%)
Mean age (SD*)	48.8 (5.6)	48.3 (5.3)
**Marital status**		
Currently married/Cohabitating	91.2%	46.1%
Never married/Cohabitated	1.5%	6.6%
Divorced/Separated/Widowed	7.3%	47.3%
Mean number of pregnancies (SD)		4.9 (2.5)
**Highest level of education**		
No formal education	3.8%	10.7%
Primary	50.5%	62.8%
Secondary	43.2%	26.1%
Tertiary	2.5%	0.4%
**Employment**		
Employed	97.4%	91.5%
Unemployed	2.6%	8.5%
**Household wealth quintile**		
First quintile	10.6%	13.4%
Second quintile	19.0%	25.0%
Third quintile	23.6%	22.8%
Fourth quintile	19.4%	21.2%
Fifth quintile	27.4%	17.6%
Household density (SD)	2.3 (2.2)	3.2 (2.3)
**Behavioural**		
**Tobacco use**		
Never smoke	46.0%	91.4%
Current smoker	22.8%	2.2%
Former smoker	27.2%	4.9%
Snuff use	3.5%	1.1%
Chewing tobacco use	0.5%	0.4%
**Alcohol consumption**		
Never consumed	29.0%	71.8%
Current non-problematic	19.6%	3.0%
Current problematic	14.2%	2.8%
Former consumer	37.1%	22.4%
Mean MVPA** (minutes per week) (SD)	1966.6 (1780.7)	1566.7 (1669.8)
Mean Sedentary Time (minutes per day) (SD)	872.7 (453.0)	868.9 (452.5)
Mean Sleep (average hours per night) (SD)	7.8 (1.5)	8.0 (1.5)
**Diet**		
Vegetable servings/day, Mean (SD)	3.9 (2.5)	4.3 (2.6)
Fruit servings/day, Mean (SD)	1.5(0.8)	1.5 (0.9)
Bread in slices/day, Mean (SD)	3.0 (2.3)	2.5 (2.0)
Eating-Out or from Vendor/week, Mean times (SD)	2.2 (2.4)	1.5 (1.9)
Number of sugary Drink/week, Mean (SD)	0.8 (2.6)	0.5 (1.1)
**Biological**		
**Ethnicity**		
Kikuyu	26.1%	44.2%
Kamba	21.9%	18.0%
Luhya	22.9%	14.8%
Luo	19.8%	13.4%
Other	9.4%	9.7%
**Self-reported diabetes**		
No	98.0%	96.0%
Yes	2.0%	4.1%
**Tested HIV positive**		
No	94.0%	83.8%
Yes	7.0%	16.2%
**Reported tuberculosis infection**		
No	88.8%	89.3%
Yes	11.2%	10.7%
**Menopausal status**		
Premenopause		43.8%
Perimenopause		17.1%
Postmenopause		39.1%
**Outcome variable**		
Mean height (m) (SD)	1.7 (0.1)	1.6 (0.1)
Mean weight (kg) (SD)	65.7 (12.0)	69.5 (15.5)
Mean BMI*** (kg/m^2^)-mean (SD)	22.8 (3.9)	27.6 (6.1)
Median BMI (kg/m^2^) (IQR****)	22.2 (21.9, 22.5)	26.9 (26.4, 27.3)
**BMI categories**		
Underweight	11.7%	3.9%
Healthy weight	63.5%	33.1%
Overweight	19.6%	30.9%
Obese	5.1%	32.1%

Notes: *SD- standard deviation, **MVPA-Moderate to Vigorous Physical Activity, ***BMI- Body Mass Index, ****IQR- Interquartile range

### Distribution of BMI by sex and ethnicity

Overall mean BMI was 25.4, but women had higher mean BMI than men (27.6 versus 22.8; p < 0.001). The prevalence values for overweight, obesity and underweight were 25.8% and 19.8% and 7.5% respectively; more women than men were overweight (38.9% versus 19.6%; p < 0.001) and obese (32.1% versus 5.1%; p < 0.01) while 11.7% of men were underweight compared to 3.9% of women who were underweight.

**Figure 2.**
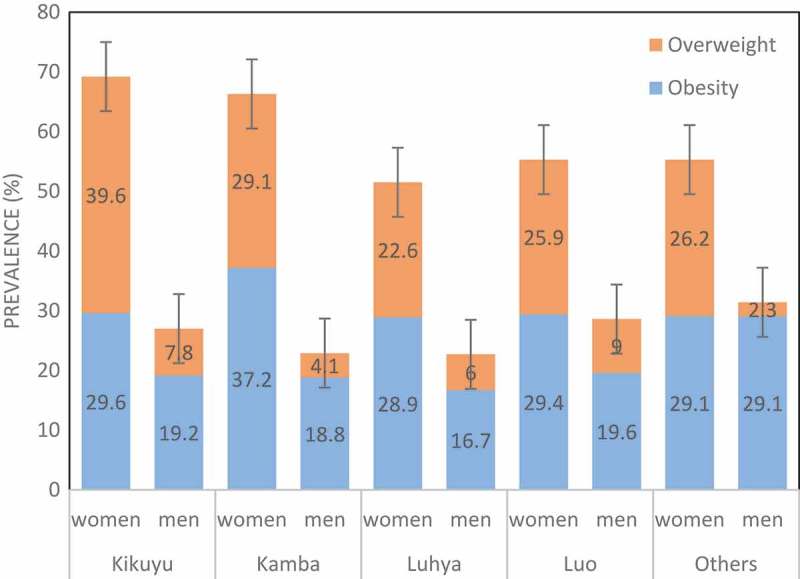
Prevalence of overweight and obesity by ethnicity among men and women aged 40–60 years in the Nairobi slums (2015–2016).

As shown in Figure 2, overweight and obesity varied by ethnicity and sex. Among women, the prevalence of obesity and overweight combined was fairly similar among the Kikuyu and Kamba but higher than that among all other ethnic groups. Among men there was a negligible difference in the obesity and overweight combined prevalence by ethnicity. Obesity alone was highest among Kikuyu and Kamba women, while among men obesity was highest among Kikuyu and the Luo.

### Factors associated with BMI

In univariate analysis, BMI among men was positively associated with being currently married or living with a partner, higher wealth quintile, self-reported diabetes, sedentary time, vegetable intake, consumption of bread and taking sugary drinks. BMI among men was inversely associated with being a current problematic drinker. Among women, age, being married or living with a partner, higher wealth quintile, diabetes, sedentary time, and number of sugary drinks were positively associated with BMI but BMI was lower in all other ethnicities compared to Kikuyu, among those currently smoking tobacco, current problematic alcohol consumers, those with longer night sleep, tuberculosis and HIV infections (Table 2).

**Table 2.** Factors associated BMI among men and women in Nairobi slums (Univariate analysis).

Characteristics	Men Coefficient (95% CI)	Women Coefficient (95% CI)
**Sociodemographic**		
Age	0.001 (−0.001, 0.003)	0.003 (0.001, 0.006)
**Marital status**		
Never married or co-habited (REF*)		
Married/Cohabitating	0.116 (0.026, 0.207)	0.076 (0.022, 0.131)
Divorced/Widowed	0.032 (−0.066, 0.130)	0.019 (−0.035, 0.073)
**Highest level of education**		
No formal education (REF)		
Primary	−0.002 (−0.060, 0.056)	0.015 (−0.029, 0.059)
Secondary	0.018 (−0.040, 0.077)	0.020 (−0.028, 0.068)
Tertiary	−0.004 (−0.093, 0.086)	0.047 (−0.171, 0.265)
**Employment**		
Unemployed (REF)		
Employed	0.014 (−0.055, 0.083)	0.023 (−0.024, 0.071)
**Wealth quintile**		
First Quintile (REF)		
Second Quintile	0.085 (0.045, 0.126)	0.067 (0.024, 0.110)
Third Quintile	0.103 (0.064, 0.142)	0.115 (0.072, 0.159)
Fourth Quintile	0.131 (0.091, 0.172)	0.171 (0.127, 0.215)
Fifth Quintile	0.152 (0.114, 0.190)	0.202 (0.156, 0.248)
Household density (persons/bedrooms)	−0.001 (−0.008, 0.006)	−0.007 (−0.015, 0.001)
**Behavioural**		
**Tobacco use**		
Never smoked (REF)		
Current smoker	−0.119 (−0.146, 0.092)	−0.208 (−0.298, −0.119)
Former smoker	−0.004 (−0.030, 0.021)	−0.013 (−0.073, 0.048)
Snuff use	−0.036 (−0.094, 0.023)	−0.025 (−0.149, 0.098)
Chewing tobacco use	−0.086 (-.244, .071)	−0.027 (−0.240, 0.187)
**Alcohol use**		
Never consumed (REF)		
Current non-problematic	−0.066 (−0.097, 0.034)	0.033 (−0.045, 0.110)
Current problematic	−0.099 (−0.133, −0.064)	−0.081 (−0.161, −0.002)
Former consumer	−0.003 (−0.030, 0.023)	−0.004 (−0.036, 0.028)
**Physical activity**		
MVPA** (minutes per week)	−0.001(−0.001, 0.001)***	0.001 (−0.001, 0.001)***
Sleep (average hours per night)	−0.014 (−0.021, 0.007)	−0.022 (−0.031, 0.013)
Sedentary Time (minutes per day)	0.003 (0.001, 0.001)***	0.001 (0.001, 0.001)***
**Diet**		
Vegetable intake (servings/week)	0.006 (0.002, 0.011)	0.002 (−0.003, 0.007)
Fruit intake (servings/week)	−0.006 (−0.019, 0.007)	0.010 (−0.005, 0.025)
Bread (slices/week)	0.008 (0.003, 0.013)	0.001(−0.006, 0.007)
Eating-out or from vendor (times/week)	−0.002 (−0.007, 0.002)	−0.002 (−0.009, 0.005)
Sugary drink (number/week)	0.005 (0.001, 0.009)	0.012 (0.0001, 0.024)
**Biological**		
**Ethnicity**		
Kikuyu (REF)		
Kamba	−0.016 (−0.047, 0.016)	−0.037 (−0.074, −0.001)
Luo	−0.005 (−0.036, 0.026)	−0.093 (−0.132, −0.053)
Luhya	0.011 (−0.021, 0.044)	−0.076 (−0.116, −0.035)
Others	0.015 (−0.027, 0.057)	−0.070 (−0.116, −0.024)
**Self-reported diabetes**		
No (REF)		
Yes	0.113 (0.034, 0.193)	0.071 (0.004, 0.139)
**Tested HIV positive**		
No (REF)		
Yes	−0.066 (−0.108, 0.024)	−0.123 (−0.158, −0.088)
**Reported tuberculosis infection**		
No (REF)		
Yes	−0.091 (−0.125, 0.056)	−0.107 (−0.149, −0.065)
**Number of pregnancies**		−0.001 (−0.007, 0.004)
**Menopausal status**		
Pre-menopause (REF)		
Peri-menopause		0.006 (−0.032, 0.044)
Menopause		0.007 (−0.022, 0.036)

Notes: *REF- reference group against which other variable categories were compared, **MVPA- Moderate to Vigorous Physical Activity, *** values corrected to 3 decimal places from very low values

After hierarchical modelling, we found that among men, sociodemographic factors contributed 7.5% of the BMI variance with wealth quintile as the main factor associated with BMI, behavioural factors (mainly current smoking, bread consumption) contributed additional 7.4% of variability in BMI and biological factors (HIV infection, reported tuberculosis, self-reported diabetes) accounted for additional 3.6% of the variance in BMI. BMI was independently associated with wealth status, bread consumption and self-reported diabetes and was negatively associated with current tobacco smoking, HIV and TB infections. All factors combined accounted for 19.6% of the variability in BMI (Table 3).

**Table 3.** Factors associated with BMI among men aged 40–60 years in Nairobi slums (2014–2015).

	Model 1		Model 2		Model 3	
Characteristics	Coeff	(95% CI)	Coeff	95% CI	Coeff	95% CI
**Sociodemographic**						
**Wealth quintile**						
First Quintile (REF)						
Second Quintile	0.074	0.034, 0.115	0.056	0.017, 0.096	0.063	0.020, 0.107
Third Quintile	0.090	0.050, 0.130	0.068	0.030, 0.107	0.063	0.021, 0.104
Fourth Quintile	0.109	0.068, 0.150	0.089	0.049, 0.129	0.083	0.039, 0.126
Fifth Quintile	0.138	0.098, 0.177	0.100	0.061, 0.139	0.092	0.050, 0.133
**Behavioural**						
**Tobacco consumption**						
Never smoked (REF)						
Current smoker			−0.084	−0.113,-0.055	−0.087	−0.120, −0.054
Former smoker			0.003	−0.025, 0.030	0.021	−0.010, 0.052
Snuff use			−0.038	−0.096, 0.020	−0.036	−0.098, 0.026
Chewing tobacco use			−0.104	−0.258, 0.050	−0.05	−0.200, 0.100
**Diet**						
Vegetable servings/week			0.001	−0.000, 0.001		
Bread slices/week			0.001	0.000, 0.002	0.001	0.001, 0.003
Sugary drinks/week			0.003	−0.001, 0.007		
**Biological**						
Self-reported Diabetes					0.099	0.023, 0.175
Tested HIV positive					−0.066	−0.113, −0.019
Reported tuberculosis					−0.082	−0.118, −0.045
R^2^	0.075		0.16		0.196	
∆R^2^			0.074		0.036	
F (p-value)	<0.001		<0.001		<0.001	
BIC	−702.152		−719.666		−561.063	

Notes: Only statistically significant factors in the final model are shown in the table. Other factors included are shown in the models: *Model 1: Sociodemographic factors (Age, marital status, wealth index); Model 2: Model 1+ behavioural factors (Smoking, Alcohol, Diet); Model 3: Model 2+ Biological factors (Ethnicity, Diabetes, HIV, Tuberculosis)*

Coeff = Coefficients

Among women, sociodemographic factors (wealth quintile) accounted for 10.4% of the variance in BMI, behavioural factors (current non-problematic drinking, current smoking, and sedentary time) contributed only an additional 2% of BMI variance and biological factors accounted for additional 6.5% of variance in BMI. BMI was positively associated with wealth, current non-problematic drinking, and sedentary time, while BMI was negatively associated with all ethnicities compared to Kikuyu, being a current smoker, duration of sleep, HIV and tuberculosis infections. The factors significantly associated with BMI explain 18.9% of the variability in BMI (Table 4).

**Table 4.** Factors associated with BMI among women aged 40–60 years in Nairobi slums (2014–2015).

	Model 1		Model 2		Model 3	
Characteristics	Coeff	95% CI	Coeff	95% CI	Coeff	95% CI
**Sociodemographic**						
**Household SES**						
First Quintile (REF)						
Second Quintile	0.061	0.020, 0.103	0.049	0.007, 0.091	0.053	0.008, 0.097
Third Quintile	0.109	0.065, 0.151	0.101	0.058, 0.144	0.089	0.043, 0.135
Fourth Quintile	0.164	0.120, 0.208	0.146	0.103, 0.190	0.131	0.084, 0.177
Fifth Quintile	0.188	0.142, 0.234	0.172	0.126, 0.219	0.144	0.095, 0.193
**Behavioural**						
**Tobacco use**						
Never smoked (REF)						
Current smoker			−0.158	−0.246, −0.071	−0.175	−0.264, −0.087
Former smoker			0.013	−0.047, 0.074	0.017	−0.046, 0.079
Snuff use			0.005	−0.114, 0.124	−0.018	−0.140, 0.103
Chewing tobacco use			−0.016	−0.220, 0.188	0.029	−0.250, 0.308
**Alcohol consumption**						
Never consumed (REF)						
Current non-problematic			0.075	0.003, 0.148	0.078	0.004, 0.151
Current problematic			−0.017	−0.096, 0.062	−0.028	−0.110, 0.054
Former consumer			0.01	−0.022, 0.041	0.009	−0.024, 0.041
Average sleep time per night			−0.015	−0.023, −0.006	−0.012	−0.021, −0.003
Sedentary time (minutes/day			0.001	0.001, 0.001	0.001	0.001, 0.001
**Biological**						
**Ethnicity**						
Kikuyu (REF)						
Kamba					−0.043	−0.079, −0.007
Luo					−0.070	−0.110, −0.030
Luhya					−0.072	−0.113, −0.031
Others					−0.085	−0.131, −0.038
HIV infection					−0.101	−0.138, −0.064
Tuberculosis infection					−0.077	−0.120–0.034
R2	0.104		0.124		0.189	
∆R2			0.02		0.065	
F (p-value)	<0.001		<0.001		<0.001	
BIC	−283.211		−266.763		−240.352	

Only statistically significant factors in the final model are shown in the table. Other factors included are shown in the models.*Model 1: Sociodemographic factors (Age, marital status, wealth index): Model 2: Model 1+ behavioural factors (Smoking, Alcohol, Diet) Model 3: Model 2+ Biological factors (Ethnicity, Diabetes, HIV, Tuberculosis).*

## Discussion

Our study found a considerably higher BMI among women than men. The prevalence of obesity was 32% among women compared to 5% among men. Obesity prevalence has doubled since the last survey conducted in the same study area in 2009 [9]. However, the difference in BMI could be due to a higher mean age (48.5 years) of our study population compared to 42 years for the study conducted a decade ago. Studies in other East African cities such as Addis Ababa in Ethiopia, Kampala in Uganda, and Dares Salaam in Tanzania found prevalence of obesity and overweight in the same range [23–25]. The prevalence of overweight in the current study is comparable to that reported among urban residents in the 2015 Kenya National NCD Steps Survey, but higher than for rural Kenyans (25% versus 16%); obesity is three times higher in our urban sample compared to that in rural Kenya (20% versus 7%) [7]. This finding is consistent with cross sectional analysis of Demographic and Health Survey data from 38 low and middle income countries which revealed higher BMI among adults in urban than in rural areas [26]. The urban-rural divide in BMI prevalence may be explained by a dietary shift from traditional diets to processed, energy-rich food, fat, animal-source foods, sugar and sweetened beverages that are in abundance in urban areas.

Higher socio-economic status in urban areas is cited as the main factor associated with higher obesity prevalence [26]. Indeed in our study, among both men and women. BMI increased with wealth as consistently reported in low- and middle-income countries [27]. Wealth index was the most significant predictor appearing in all the models and influencing other risk factors for BMI. This may be due to an increased capacity to access high-energy dense foods which are often imported and generally more expensive. The ownership of cars and ability to afford motorized transport and employing domestic workers to undertake labour intensive household chores are associated with less energy expenditure among wealthier people and could contribute to higher BMI values among wealthier individuals.

However, there was a substantial independent contribution of behavioural factors to BMI especially among men, where we observed consumption of bread to be associated with higher BMI. Men consumed three slices of bread per day on average slightly higher than women who consumed about two slices per day. A cohort study in Spain showed an association between weight gain and consumption of white bread but not with whole meal bread [28]. White bread is cheaper than brown or whole meal bread and thus affordable and accessible to participants in the study regardless of socioeconomic status.

Among women, behavioural factors had a minimal contribution to BMI. Sedentary life was the main factor associated with BMI as published elsewhere [29]. However, in this study, physical activity was not associated with BMI. This is to emphasize that sedentary behaviour and physical activity are different constructs contributing to BMI through independent pathways, and not necessarily as opposing ends of the same spectrum. In most countries, women are increasingly taking up more sedentary occupations resulting in a rise in prevalence of obesity among women working in non-agricultural occupations [30]. Most women in the slums of Nairobi are self-employed engaging in businesses which often require prolonged sitting.

We found some behavioural factors to be associated with lower BMI. Both men and women who reported current smoking had lower BMI compared to non-smokers. This association was reported before [31]. One plausible biological explanation advanced is that nicotine in tobacco increases metabolic activity by increasing adrenaline and other metabolic hormones [32], thus leading to weight loss. However, it is important to emphasize that both smoking and obesity are common risk factors for NCDs. Tobacco smoking cannot therefore be used as a measure to prevent obesity.

Our study also found that among women, longer hours of night sleep was associated with lower BMI as published before [33]. It has been postulated that acute deprivation of sleep leads to changes in leptin and ghrelin levels, and increases hunger and appetite [34]. Another study reported a decreased physical activity due to sleep loss as responsible for weight gain [35]. Our study only found this association among women. This may be explained by sex hormone changes during menopause associated with weight gain and decreased sleep [36].

Women from other ethnic groups in the study had lower BMI compared to the Kikuyu women. Similar associations of BMI and ethnicity have been reported elsewhere even after controlling for wealth and other behavioural factors [37]. This may be explained in part by genetic differences and unmeasured socio-cultural differences in dietary habits among women of various ethnic groups that will require further investigation.

It was not surprising to find diabetes to be associated with higher BMI among men since BMI is a known risk factor for diabetes from longitudinal studies [38]. HIV and Tuberculosis infections are known to cause weight loss through various mechanisms [39,40], thus the lower BMI among men and women who had HIV positive results and self-reported tuberculosis infections in our study population.

The strength of this study lies in availability of a large sample that allowed us to examine risk factors for high BMI by gender. The study was embedded in NUHDSSs where deep relationships were built with the communities over many years. The choice of this study site is a potential limitation itself since participants in the NUHDSS have been involved in several studies and this could lead to social desirability bias associated with under reporting of undesirable behaviours. For most of the variables we relied on a self-report, which could have particularly affected the validity of questions on lifestyle factors such as dietary questions, physical activity, use of alcohol and tobacco. However, we used standard score cards to guide respondents on estimating quantities of food or alcohol consumed and conducted precision estimates among responses from various field workers to ensure that intra- and inter- coefficient of variations were below 2–5%.

## Conclusions

A substantial proportion of the population in the urban slums in Nairobi have a high BMI with women having a higher BMI than men. This study provided an opportunity to understand independent factors associated with a high BMI among men and women to guide gender targeted interventions. Wealth was associated with high BMI among both men and women. Behavioural factors such as bread consumption among men, sedentary life and non-problematic drinking among women were associated with a higher BMI. These findings highlight unhealthy behaviours typical of urban culture. Some behavioural factors including tobacco smoking among women and men, longer night hours of sleep among women were associated with lower BMI. Among biological factors assessed, self-reported diabetes among men was associated with a higher BMI while HIV infection and reported tuberculosis infection among both men and women were associated with lower BMI. Women belonging to all ethnic groups had lower BMI compared to women of Kikuyu ethnicity. Tailored messages for men and women with emphasis on the different lifestyles are needed. There is need to engage with policy makers and program managers to promote healthy eating habits, irrespective of wealth status, and discourage sedentary lifestyle and alcohol consumption among women to address the high burden of obesity in the slums. A multi-sectoral approach involving urban planners, ministry of health, agriculture, and academia among other sectors will be very crucial in promoting policies for preventing obesity in the slums.

## Paper context

High body mass index is a known risk factor for all the four main non-communicable diseases and is rising disproportionately in urban areas in low and middle-income countries and higher among women. It is crucial to identify gender specific factors driving BMI increase to appropriately tailor interventions. Indeed, this study found, besides wealth, different unhealthy behaviours for men (excessive bread consumption) and women (sedentary life, alcohol consumption). Multi-stakeholder engagement is required to design targeted interventions.
